# Supplementary material for: Anchor-Based and Distributional Responsiveness of the Spanish Version of the Edinburgh Feeding Evaluation in Dementia Scale in Older People with Dementia: A Longitudinal Study
Source: Nutrients. 2024 Nov 12;16(22):3863. doi: 10.3390/nu16223863 (PMC11597151; doi:10.3390/nu16223863)
Supplement: Supplementary file 1 [file nutrients-16-03863-s001.zip › File S1. Spanish version of the EdFED scale.pdf]

File S1 (Spanish version of the EdFED)

Edinburg Feeding Evaluation in Dementia Scale (EdFED) (Roger Watson, 1993ayb) (1,2)

Versión validada al español (Saucedo Figueredo MC, 2016 y 2018) (3,4)

EdFED

	Nunca (0)	A veces (1)	A menudo (2)
1. ¿Requiere el paciente estrecha supervisión durante la alimentación?	☐	☐	☐
2. ¿Requiere el paciente ayuda física con la alimentación?	☐	☐	☐
3. ¿Se le derrama la comida mientras se alimenta?	☐	☐	☐
4. ¿Tiende el paciente a dejar comida en el plato al final de la comida?	☐	☐	☐
5. ¿Se niega el paciente alguna vez a comer?	☐	☐	☐
6. Vuelve el paciente la cabeza hacia otro lado mientras se le alimenta?	☐	☐	☐
7. ¿Se niega el paciente a abrir la boca?	☐	☐	☐
8. ¿Escupe el paciente su comida?	☐	☐	☐
9. ¿Deja el paciente la boca abierta permitiendo que la comida se caiga fuera?	☐	☐	☐
10. ¿Se niega el paciente a tragar?	☐	☐	☐

11. Indique el nivel adecuado de cuidado para la alimentación que requiere el paciente:

☐ Apoyo-educativo

☐ Parcialmente compensatorio

☐ Totalmente compensatorio

1. Watson R, Deary IJ. Measuring feeding difficulty in patients with dementia: multivariate analysis of feeding problems, nursing intervention and indicators of feeding difficulty. Journal of Advanced Nursing. 1 de agosto de 1994;20(2):283-7.
2. Watson R. Estimating the relative level of feeding difficulty in older patients with dementia. University of Sussex, Brighton. 1993b;
3. Saucedo Figueredo MC, Morilla Herrera JC, Ramos Gil R, Arjona Gómez MN, García Dillana F, Martínez Blanco J, et al. Validation of the Spanish version of the Edinburgh feeding evaluation in dementia scale applied to institutionalized older persons with dementia: a study protocol. Nurs Open. 1 de marzo de 2016;236-42.
4. Saucedo Figueredo MC, Morilla Herrera JC, San Alberto Giraldos M, López Leiva I, León Campos Á, Martí García C, et al. Validation of the Spanish version of the Edinburgh Feeding Evaluation in Dementia Scale for older people with dementia. PLoS ONE. 2018;13(2):e0192690.
