# Supplementary material for: Anchor-Based and Distributional Responsiveness of the Spanish Version of the Edinburgh Feeding Evaluation in Dementia Scale in Older People with Dementia: A Longitudinal Study
Source: Nutrients. 2024 Nov 12;16(22):3863. doi: 10.3390/nu16223863 (PMC11597151; doi:10.3390/nu16223863)
Supplement: Supplementary file 1 [file nutrients-16-03863-s001.zip › File S2. English EdFED scale.pdf]

File S2 (English EdFED scale)

Edinburgh Feeding Evaluation in Dementia Scale (EdFED) (Roger Watson, 1993ayb) (1, 2)

Item	Never	Sometimes	Often
1. Does the patient require close supervision while feeding?	□	□	□
2. Does the patient require physical help with feeding?	□	□	□
3. Is there spillage while feeding?	□	□	□
4. Does the patient tend to leave food on the plate at the end of a meal?	□	□	□
5. Does the patient ever refuse to eat?	□	□	□
6. Does the patient turn his head away while being fed?	□	□	□
7. Does the patient refuse to open his mouth?	□	□	□
8. Does the patient spit out his food?	□	□	□
9. Does the patient leave his mouth open allowing food to drop out?	□	□	□
10. Does the patient refuse to swallow?	□	□	□

11. Indicate the appropriate level of care for feeding required by patient:

□ supportive-educative

□ partly compensatory

□ wholly compensatory

1. Watson R. Measuring feeding difficulty in patients with dementia: perspectives and problems. J Adv Nurs. Enero de 1993;18(1):25-31.
2. Watson R. Estimating the relative level of feeding difficulty in older patients with dementia. University of Sussex, Brighton; 1993.
