# Supplementary material for: Anchor-Based and Distributional Responsiveness of the Spanish Version of the Edinburgh Feeding Evaluation in Dementia Scale in Older People with Dementia: A Longitudinal Study
Source: Nutrients. 2024 Nov 12;16(22):3863. doi: 10.3390/nu16223863 (PMC11597151; doi:10.3390/nu16223863)
Supplement: Supplementary file 1 [file nutrients-16-03863-s001.zip › File S3. Variables Table.pdf]

File S3: Measurement variables Table

Instrument/Tool Name	Variable Type	Possible Score	Reference Values
Functional evaluation (Barthel index "BI") (1)	Continuous quantitative	0-100 point	• 0-20 Total physical dependence<br>• 21-60 Severe physical dependence<br>• 61-90 Moderate physical dependence<br>• 91-99 Little physical dependence<br>• 100 Physically independent
Mental Status<br>Pfeiffer Questionnaire "PQ" (2)	Discrete quantitative	0-10 point	• 0-2 Normal<br>• 3-4 Mild cognitive impairment<br>• 5-7 Moderate cognitive impairment<br>• 8-10 Severe cognitive impairment
Severity of Dementia<br>(GDS Fast)	Discrete quantitative	1-7 point	• 1 Absence of cognitive deficit<br>• 2 Very mild cognitive deficit<br>• 3 Mild cognitive deficit<br>• 4 Moderate cognitive deficit<br>• 5 Moderately

			severe cognitive deficit<br>• 6 Severe cognitive deficit<br>• 7 Very severe cognitive deficit
Comorbidity<br>Charlson Comorbidity Index "CCI" (3)	Discrete quantitative	1-37 point.<br><br>Prediction of mortality in short follow-ups (< 3 years);<br><br>index of 0: (12% mortality/year); index 1-2: (26%); index 3-4: (52%); index > 5: (85%)	• 0-1 point: Absence of comorbidity<br>• 2 points: Low comorbidit<br>• 3 or more: High comorbidity
Height (cm.) *	Continuous quantitative	0-200 point	Carbon fiber height meter or tape measure
Weight (Kg.) **	Continuous quantitative	0-130 point	Analogy numerical scale
Nutritional Status:<br>Body Mass Index (BMI) % (Older Adjusted Values) (4)<br>(%)=Weight(kg.)/Height(m2)	Continuous quantitative	<16>29.9%	• 29.9-23% Normal weight<br>• 22.9-18.5% Insufficient weight<br>• 18.4-17% Mild malnutrition<br>• 16.9-16% Moderate malnutrition<br>• <15.9 Severe malnutrition
Nutritional Status<br>(Mini Nutritional Assessment "MNA") (5)	continuous quantitative	<17>23.5 points	• >23.5 points: satisfactory status.<br>• 17 to 23.5 points:

			risk of malnutrition.<br>• < 17 points: poor nutritional status
Muscle Mass<br>(Forearm Circumference "FC") (cm.) (5)	continuous quantitative	<21cm.>22cm.	Carbon fiber measuring tape.<br>• CB < 21 cm. Malnutrition<br>• 21cm. <CB < 22cm. Risk of Malnutrition<br>• CB > 22 cm. Normal
Muscle Mass<br>(Calf Circumference "CC") (cm.) (5)	continuous quantitative	<31cm.≥31cm.	Carbon Fiber Tape Measure<br>• CP < 31cm Malnutrition<br>• CP≥ 31 cm. Normal
Lymphocytes (%)***	continuous quantitative	<20-53>	Blood analysis test<br>• <20 Risk of malnutrition<br>• 20-53 Normal nutrition<br>• >53 altered
Absolute lymphocytes Cel./mm3.***	Continuous quantitative	800-2.000 cel./mm3.	Blood analysis test<br>• 2.000 cel./mm3- 1.800 cel./mm3 Normal<br>• 1.799 cel./mm3- 1.200 cel./mm3 Mild Malnutrition<br>• 1.199 cel./mm3- 800 cel./mm3 Moderate Malnutrition

			• <800 cel./mm3 Severe Malnutrition
Total Proteins g./dl. ***	Continuous quantitative	<5 g/dl.≥	Blood analysis test<br>• ≥5 g./dl. Low risk of malnutrition<br>• <5 g./dl. Medium risk of malnutrition
Cholesterol mg. /dl. ***	Continuous quantitative	100-180 mg./dl.	Blood analysis test<br>• >180 mg./dl. Normal<br>• 180 mg./dl-140 mg./dl. Mild Malnutrition<br>• 139 mg./dl.-100 mg./dl. Moderate Malnutrition<br>• <100 mg./dl Severe Malnutrition
Albumin g. /dl. ***	continuous quantitative	<2.1g./dl.>5.3 g./dl.	Blood analysis test<br>• 5.3 g/dl g./dl.-3.6 g./dl. Normal<br>• 3.5g./dl.-2.8 g./dl. Mild Malnutrition<br>• 2.7 g./dl.-2.1 g./dl. Moderate Malnutrition<br>• <2.1 g./dl Severe Malnutrition
Transferrin mg. /dl. ***	Continuous quantitative	100-300 mg. /dl.	Blood analysis test<br>• 300 mg./dl.-176 mg. /dl. Normal<br>• 175 mg. /dl.-150 mg. /dl. Mild Malnutrition

			• 149 mg. /dl.-100 mg. /dl. Moderate Malnutrition<br>• <100 mg. /dl. Severe Malnutrition
Feeding difficulties (EdFED scale)	Discrete quantitative	0-22 points (Frequencies “never, sometimes. often")<br>There is no cut-off point, the higher the score, the more difficulties with feeding.	• Never=0<br>The behavior is never observed)<br>• Sometimes=1<br>The behavior appears 2-3 times<br>• Often=2<br>The behavior appears 4 or more times

* In people who cannot be sized (bedridden or with a deformity), the alternative anthropometric measurement tables (6), will be used, according to knee height.

.

** In people who cannot be sized (bedridden or with a deformity), the alternative anthropometric measurement tables (6), depending on forearm length, knee height and age.

*** Reference values cited in; Consensus document between the SENPE and the SEGG. Nutritional assessment in the elderly (7).

Measurement variables Table: where all the tools used for data collection are described

Bibliography

1. Mahoney FI, Barthel DW. Functional Evaluation: The Barthel Index. Md State Med J. febrero de 1965;14:61-5.

2. Pfeiffer E. A short portable mental status questionnaire for the assessment of organic brain deficit in elderly patients. J Am Geriatr Soc. octubre de 1975;23(10):433-41.

3. Charlson ME, Pompei P, Ales KL, MacKenzie CR. A new method of classifying prognostic comorbidity in longitudinal studies: development and validation. J Chronic Dis. 1987;40(5):373-83.

4. Salas Salvadó J, Rubio MA, Barbany M, Moreno B, Grupo Colaborativo de la SEEDO. Consenso SEEDO 2007 para la evaluación del sobrepeso y la obesidad y el establecimiento de criterios de intervención terapéutica. Med Clin (Barc). 2007;128(5):184-96.

5. Guigoz Y, Vellas B, Garry PJ. Mini Nutritional Assessment: a practical assesment tool for grading the nutricional state of elderly patients. Facts and Research in Gerontology. 1994;12(Supl 2):15-59.

6. Rabat Restrepo JM, Rebollo Pérez I. Medidas antropométricas alternativas. Proceso de Soporte de Nutrición Clínica y Dietética. Consejería de Salud de Andalucía. [Internet]. SANCYD; 2004. Disponible en: http://www.sancyd.es/luis/tablas/99032.pdf

7. García de Lorenzo y Mateos A, Ruipérez Cantera I. Valoración Nutricional en el anciano [Internet]. SENPE y SEGG; 2007. Disponible en: http://www.senpe.com/IMS/publicaciones/consenso/senpe_valoracion_nutricional_anciano.pdf
